# Foot strength and stiffness are related to footwear use in a comparison of minimally- vs. conventionally-shod populations

**DOI:** 10.1038/s41598-018-21916-7

**Published:** 2018-02-27

**Authors:** Nicholas B. Holowka, Ian J. Wallace, Daniel E. Lieberman

**Affiliations:** Department of Human Evolutionary Biology, Harvard University, Cambridge, MA 02138 USA

## Abstract

The longitudinal arch (LA) helps stiffen the foot during walking, but many people in developed countries suffer from flat foot, a condition characterized by reduced LA stiffness that can impair gait. Studies have found this condition is rare in people who are habitually barefoot or wear minimal shoes compared to people who wear conventional modern shoes, but the basis for this difference remains unknown. Here we test the hypothesis that the use of shoes with features that restrict foot motion (e.g. arch supports, toe boxes) is associated with weaker foot muscles and reduced foot stiffness. We collected data from minimally-shod men from northwestern Mexico and men from urban/suburban areas in the United States who wear ‘conventional’ shoes. We measured dynamic LA stiffness during walking using kinematic and kinetic data, and the cross-sectional areas of three intrinsic foot muscles using ultrasound. Compared to conventionally-shod individuals, minimally-shod individuals had higher and stiffer LAs, and larger abductor hallucis and abductor digiti minimi muscles. Additionally, abductor hallucis size was positively associated with LA stiffness during walking. Our results suggest that use of conventional modern shoes is associated with weaker intrinsic foot muscles that may predispose individuals to reduced foot stiffness and potentially flat foot.

## Introduction

As bipeds, humans have evolved dramatically different feet from other primates^1^. One of the most distinctive features of the human foot is the longitudinal arch (LA), whose anatomical scaffold is created by the conformation of the tarsal and metatarsal bones, and which is reinforced by numerous soft tissue structures that span the plantar surface of the foot. The LA stiffens the foot under loading, enabling it to function as a propulsive lever during walking and running^2^. LA stiffness partly derives from ligamentous structures, including the long and short plantar ligaments, the spring ligament and the plantar aponeurosis, that traverse the plantar surface of the foot longitudinally and act as trusses to resist compressive forces on the LA^3^. The intrinsic foot muscles also contribute to LA stiffness by contracting to help control LA deformation during walking and running^4,5^, thereby relieving an unknown proportion of the stress borne by the plantar ligaments.

The standing height of the LA on the medial side of the foot is the most commonly used indicator of relative arch height^6^. Individuals with exceptionally low LAs while standing are characterized as having flat foot (*pes planus*). All humans are born with a low arch, and most develop a fully adult configuration of the LA by 10–12 years of life^7^. However, roughly 20–25% of adults in the United States and Canada are diagnosed as having flat feet^8–11^, either because they fail to develop a normal height arch or because the arch collapses. Most individuals diagnosed with flat foot possess a so-called ‘flexible’ flat foot, characterized by substantial eversion of the rear foot during weight-bearing, resulting in a marked drop in LA height^12^, and reduced LA stiffness during walking^13,14^. Although this condition is often asymptomatic^12^, in some individuals it causes foot pain and fatigue after long durations standing and/or walking^15^. Reduced LA stiffness is also a risk factor for numerous lower extremity musculoskeletal disorders including plantar fasciitis, knee osteoarthritis, tibialis posterior tendinopathy, and metatarsal stress fracture^11,16–19^. Thus, developing strategies to prevent and treat this condition is an important health objective.

Despite the high incidence flat feet in the US and other developed nations, many studies report lower rates of flat foot in habitually barefoot or minimally-shod populations^20–28^. In one of the largest of these studies, which included 1,846 adults from southern India, Sachithanandam and Joseph^28^ found that individuals who never wore shoes before age 16 had roughly half the rate of flat foot of those who grew up wearing shoes. More recently, in a study of 810 school children between 6 and 18 years old, Hollander *et al*.^25^ found significantly higher LAs in children who were habitually barefoot compared to those who were habitually shod. These findings are potentially significant given that, until relatively recently, all humans were either barefoot or wore minimal footwear lacking the cushioning, arch supports, restrictive toe boxes and other features of conventional shoes. It has also been shown that the use of minimal shoes by adults who grew up in conventional modern shoes is associated with increases in intrinsic foot muscle size, as well as LA height and stiffness^29–31^. It is therefore reasonable to hypothesize that the reduction in LA stiffness that characterizes flat foot is a mismatch condition caused by the human foot being inadequately adapted to the novel environmental condition of wearing shoes that provide comfort and protection at the expense of weaker foot muscles^32^. However, no study has investigated whether people who grew up habitually barefoot or wearing minimal shoes have stronger foot muscles than those who grow up wearing conventional modern shoes. Thus, the relationship between foot muscle strength, footwear use, and LA stiffness needs to be tested.

**Figure 1:**
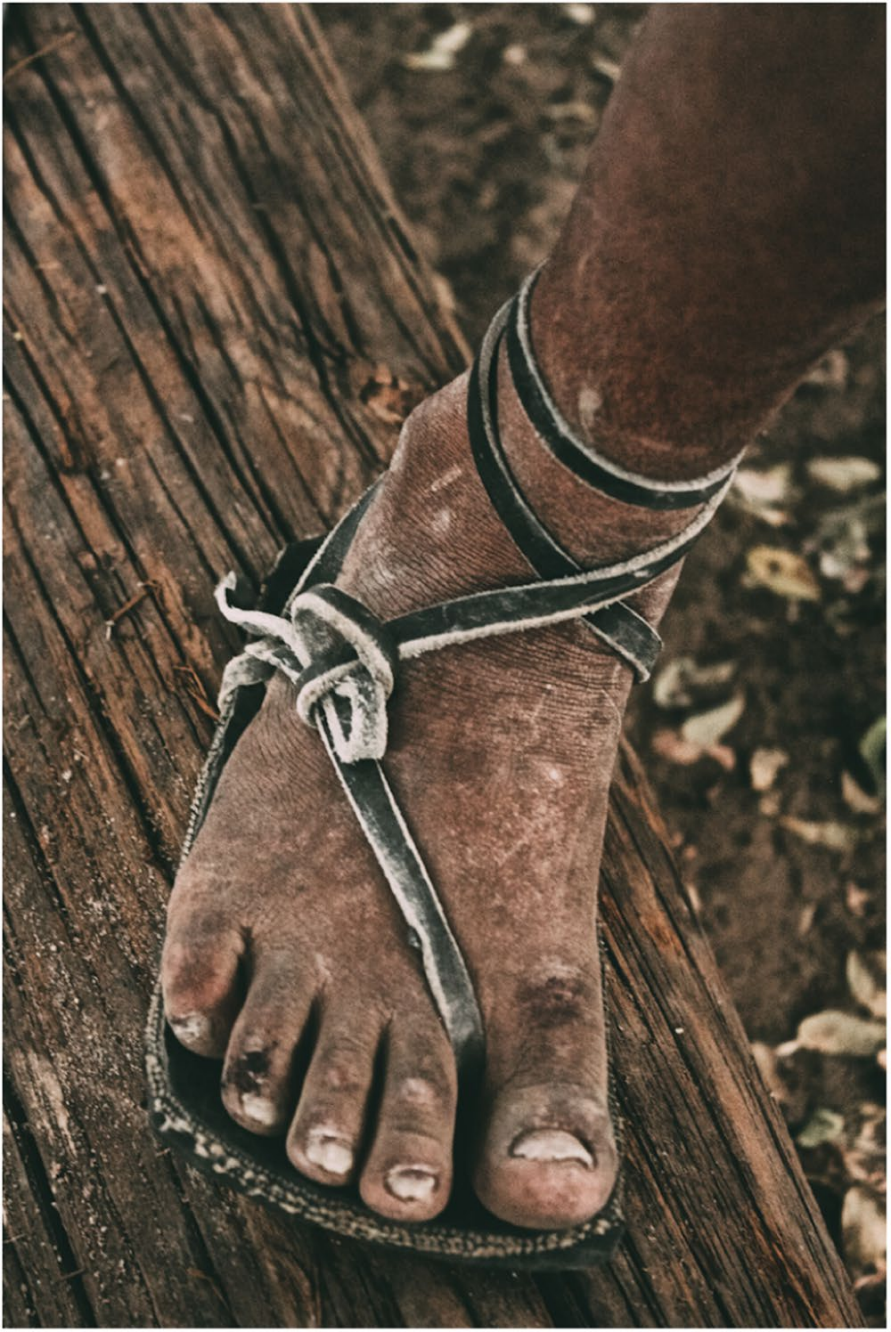
A Tarahumara man wearing a typical sandal with a sole made from car tire rubber. Photo copyright© 2018 by David Ramos and used here with permission. All rights reserved.

This study uses retrospective data as an initial test of the hypothesis that individuals who are habitually minimally-shod throughout life have stronger foot muscles and stiffer feet than those who habitually wear ‘conventional shoes’, which we define here as shoes with some combination of features that affect natural foot motion, including restrictive toe boxes, heel counters, arch supports and toe springs. Since few individuals in the United States or other developed countries grow up barefoot or minimally-shod, we compared LA stiffness and intrinsic foot muscle strength in conventionally-shod adults from the United States with age-matched adults from a minimally-shod population of Tarahumara (Rarámuri) Native Americans from the Sierra Tarahumara, a mountainous region of northwestern Mexico. Although, they have gained renown for ultra-long distance running^33^, most Tarahumara run infrequently, with most of their physical activity consisting of farming and walking long distances. During all activities, including running, they typically wear minimal sandals (*huaraches*) consisting of soles made from car tire rubber affixed to the foot and ankle by leather thongs (Fig. 1). In recent times, conventional modern footwear has become increasingly common among the younger Tarahumara, many of whom are moving from isolated farms to urban environments. A recent study found that conventionally-shod Tarahumara have significantly less stiff and lower LAs than minimally-shod Tarahumara^33^. For the present study, we predicted that minimally-shod Tarahumara men have both larger intrinsic foot muscles and stiffer LAs than men of similar ages and body sizes who have been habitually conventionally-shod most of their lives. We also predicted that intrinsic foot muscle size is positively correlated with LA stiffness across groups.

An additional hypothesis this study tested is whether static measures of LA stiffness accurately reflect the dynamic function of the foot during walking. Most previous studies of barefoot and minimally-shod populations measured only static LA height and stiffness, but any influence of LA deformation on musculoskeletal disorders occurs primarily during dynamic loading *via* mechanisms such as increased stress on plantar soft tissue structures and higher bending forces on the metatarsals^16,17^. Hollander *et al*.^25^ measured static arch height based on palpable anatomical landmarks and found that these measurements do not always correspond to dynamic arch indices measured during walking using a pedography platform. However, this result is perhaps not surprising, as McPoil and Cornwall^34^ have demonstrated that pedography-based estimates of arch height are poor predictors of LA height measurements that are based on anatomical landmarks, and thus may not accurately reflect changes in LA height in response to loading. This finding indicates the necessity of measuring of LA height and stiffness dynamically using kinematic data rather than just pedography measurements. We predicted that minimally-shod individuals have dynamically stiffer LAs during walking than conventionally-shod individuals, and that dynamic LA stiffness is positively correlated with both static LA stiffness and intrinsic foot muscle size.

## Methods

### Sample

For the minimally-shod population, we collected data from 75 Tarahumara men (mean ± SD: age, 64 ± 10 yrs; body mass, 64 ± 10 kg; height, 1.58 ± 0.06 m) from the area around the Barranca de Sinforosa in the southwestern part of the state of Chihuahua, Mexico in May and June of 2016. Participants were recruited by word of mouth with the help of members of the community. To exclude individuals who do not primarily wear sandals, we limited our recruitment to individuals 50 years or older, as many younger Tarahumara have grown up wearing shoes, and we excluded individuals who reported that they wore sandals less than five days/week in warm seasons. For the habitually conventionally-shod population, we recruited by word of mouth an age-matched sample of 26 men (mean ± SD: age, 57 ± 11 yrs; body mass, 82 ± 12 kg; height, 1.79 ± 0.08 m) from urban and suburban areas in the United States (Boston, MA, Ithaca, NY, and Dayton, OH), all of whom habitually wear conventional modern shoes. For all minimally- and conventionally-shod participants, exclusion criteria included recent foot pain, previous injury to the foot and any overt gait abnormality. Participants also completed a survey in which they estimated the average number of hours walked and run per day over the previous five years.

All U. S. participants gave their written informed consent, and all Tarahumara participants provided verbal informed consent, which was administered by translators who spoke Spanish and Rarámuri (the native language of the Tarahumara). All procedures involving U. S. and Tarahumara participants were approved by Harvard University’s Institutional Review Board, and all research was carried out in accordance with the approved guidelines and regulations.

### Anthropometrics

We measured participant height and body mass and used these to calculate Body Mass Index (BMI) as body mass/height^2^. We also measured lower limb length (distance from greater trochanter to the ground), and used a custom-machined device to measure total foot length, truncated foot length (from the heel to the first metatarsophalangeal joint) and the dorsum height at 50% of foot length in both seated and standing conditions. Arch Height Index (AHI) was calculated as the ratio of the foot’s dorsum height at 50% of foot length to the total length of the foot excluding the toes when participants were *standing*. This has been shown to be a robust and repeatable measure of LA height^6^. Following previous studies^35,36^, we classified participants as having ‘low’ LAs if their AHI values were below 0.297, which is 1.5 standard deviations below the average AHI reported in a large sample of adult males from the U.S.^6^. Arch Stiffness Index (ASI), which is a static measure of LA stiffness, was calculated using seated and standing AHI values: ASI = (body mass*0.4)/(AHI_seated_ − AHI_standing_)^37^.

### Ultrasound

**Figure 2:**
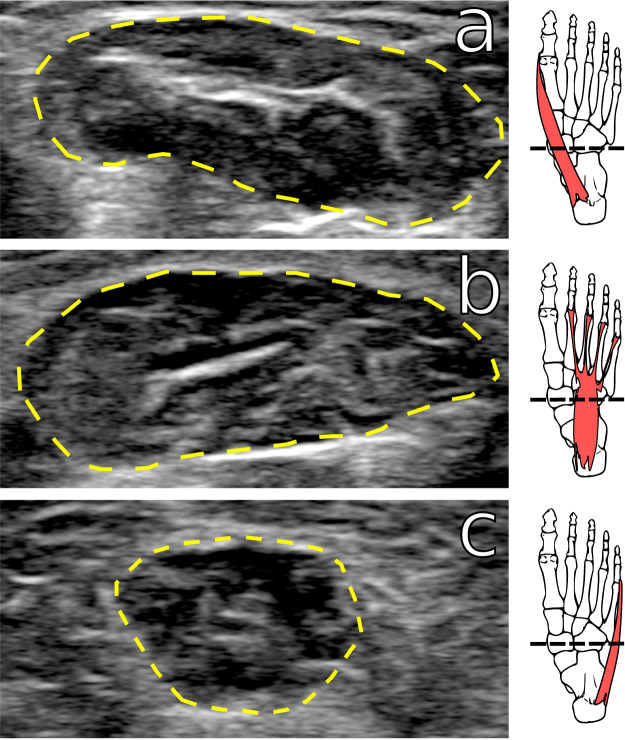
Examples of muscle cross-sectional area images taken using ultasound. (**a**) Abductor hallucis, (**b**) flexor digitorum brevis, and (**c**) abductor digiti minimi. Dashed line in foot skeleton illustrations indicates plane in which images were taken, based on palpation of the navicular tuberosity.

Cross sectional areas of the intrinsic foot muscles in the right foot were captured using a Philips L12-4 B-Mode Ultrasound Transducer (Philips Ultrasound, Inc., Bothell, WA), which has a 4–12 MHz frequency range and a 41 mm linear array. To avoid possible inter-investigator error, all ultrasound images were captured by a single, trained investigator (N.B.H.). To standardize image capture across participants, the navicular tuberosity was identified using surface palpation, and a line was drawn across the plantar surface of the participant’s foot using an ink marker to indicate the frontal plane bisecting the navicular tuberosity (Fig. 2). The ultrasound transducer was moved along this line to take frontal plane images of the foot, which were instantly recorded on a Samsung Galaxy Tablet S2 (Samsung Electronics America, Inc., Ridgefield Park, NJ) using Lumify software (Philips Ultrasound, Inc., Bothell, WA).

Ultrasound images were used to quantify the cross-sectional areas of three intrinsic foot muscles: abductor hallucis (AH; Fig. 2a), flexor digitorum brevis (FDB; Fig. 2b), and abductor digiti minimi (ADM; Fig. 2c). We measured AH and FDB because both muscles are thought to help stiffen the LA during locomotion^5,38,39^. The role of ADM in LA support is unclear^4^, but was included because Miller *et al*.^29^ found that this muscle increased size in runners who switched to training in minimal shoes. The cross-sectional areas of these muscles have been shown to be measureable using ultrasound with high intra-investigator reliability^31,40,41^. One investigator (N.B.H.) measured muscle cross-sectional area in ultrasound images using the draw tool in ImageJ^42^. To avoid potential bias in measurement, all ultrasound images were assigned random ID numbers and measured without the investigator knowing which individual or population the image came from. For some muscles in some participants, muscle boundaries were not clear due to factors such as thick callused skin that impeded ultrasound waves, and in these cases these muscles were not measured.

For statistical analyses, cross-sectional area values were scaled by dividing by (body mass)^0.67^, under the assumption of geometric similarity, where cross-sectional area ∝ (body mass)^0.67^. This relationship assumes that anatomical structures scale isometrically with respect to body size, and may be appropriate for intra-species comparisons where different individuals are expected to be geometrically similar to one another^43^. Alexander *et al*.^44^ and Myatt *et al*.^45^ have found that hind limb muscle *physiological cross-sectional area* tends to scale with a slightly higher coefficient of allometry ~ (body mass)^0.75^ in bovids and non-human great apes than predicted by geometric similarity, making this a conservative scaling metric. *Physiological cross-sectional area* differs from the cross-sectional area measurements used in this study because it is also proportional to the cosine of fiber pennation angle. However, because the muscles measured in this study all have low fiber pennation angles in humans (<20°)^46^, their physiological cross-sectional areas will be only minimally affected by pennation.

### Kinematic and kinetic data

We collected kinematic and kinetic data from all conventionally-shod participants and from a similar-sized random subsample of minimally-shod participants (N = 30; mean ± SD: age, 60 ± 8 yrs; body mass, 66 ± 12 kg; height, 1.58 ± 0.06 m). Participants walked barefoot over an Emed q-100 pedography platform (Novel GmbH, Munich, Germany) while they were being video recorded by two GoPro Hero 4 cameras (GoPro, Inc., San Mateo, CA, USA), with 7.5 mm 3MP M12 lenses (Back-Bone, Inc., Kanata, ON, Canada). One camera was positioned 0.5 m from the pedography platform to record a medial view of the participant’s right foot at a frame capture rate of 240 Hz. The second camera was positioned 2 m from the pressure platform to record a lateral view of the participant’s full body at a frame capture rate of 120 Hz. The pedography platform recorded vertical ground reaction forces at a rate of 100 Hz. Camera and platform recordings were synchronized using a light on the platform that illuminated at the instant of foot contact (‘touchdown’). The end of stance, ‘liftoff’, was determined from the medial camera video as the frame when the toes lost contact with the platform.

**Figure 3:**
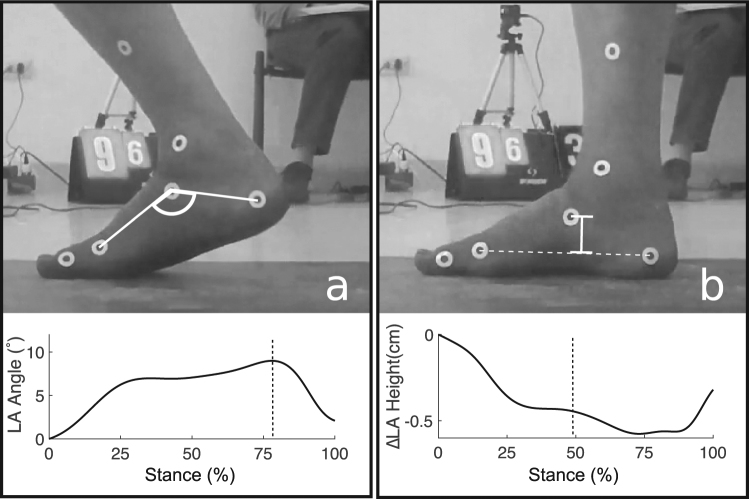
Measurements of LA kinematics during walking. (**a**) LA angle, where Maxθ was measured at the maximum value across stance as indicated by the dashed line in lower graph. (**b**) ΔLA height, where ΔLA height was measured at mid-stance, as indicated by the dashed line in the lower graph. 0° and 0 cm height are defined by marker positions at foot strike.

Prior to recording, small circular white tape markers were placed on the right lower limbs of participants. To measure LA angle, we placed markers on the medial aspect of the first metatarsal head, the navicular tuberosity, and the medial aspect of the posterior calcaneus (Fig. 3), and to measure walking speed we placed a marker on the greater trochanter. At the start of recording sessions, participants were instructed to practice walking barefoot across the pedography platform until they could touchdown near the center of the platform with the right foot while maintaining a normal gait at a constant, comfortable speed. We then recorded subjects walking for a minimum of three trials at self-selected speeds.

We digitized marker position in videos of the medial foot in MATLAB (MathWorks, Inc., Natick, MA) using the DigitizingTools_20160818 package^47^. To reduce signal artifacts caused by digitizing error, we filtered the raw marker coordinate data using a fourth order low-pass Butterworth filter with a 20 Hz cutoff frequency. We used a custom-written MATLAB routine to calculate two LA motion-related variables from the filtered data: maximum LA angle during stance (θ_max_), and mid-stance LA stiffness (k_mid_). θ_max_ was calculated as the maximum angle formed by the three markers on the medial foot during stance phase, with LA angle at touchdown set to 0°, and LA angle increasing with greater midfoot deformation (Fig. 3a). k_Mid_ was calculated using the following formula:

1 $${k}_{mid}=\frac{{F}_{mid}}{{\rm{\Delta }}LA\,height}$$

*F*_*mid*_ is the vertical ground reaction force at 50% of stance phase, which we measured with the pedography platform. To determine *ΔLA height*, we calculated LA height as the perpendicular distance between the navicular tuberosity and a line bisecting the first metatarsal head and medial calcaneus markers (Fig. 3b). We calculated *ΔLA height* as the difference between LA height at touchdown and LA height at 50% of stance phase. We calculated this LA stiffness value at 50% of stance because this is when fore-aft ground reaction forces are near 0 N, making the three-dimensional ground reaction force vector nearly perpendicular to the ground. Because the whole foot is in contact with the ground at this point in stance, the linear dimension used to measure LA height is also roughly perpendicular to the ground, and therefore parallel to the ground reaction force vector. Thus, we expect the change in LA height at mid-stance to be caused by vertical ground reaction forces, and therefore k_mid_ should reflect relative LA stiffness at this point in stance. k_mid_ was standardized by dividing by (body mass)^0.67^, under the assumption that k_mid_ should scale geometrically, as it does for limb stiffness^48^.

For each stride analyzed we measured walking speed in ImageJ^42^ using the lateral camera videos. To do so we calculated the distance travelled by the greater trochanter marker during the full stride cycle in which the foot contacted the pedography platform divided by the stride duration. To analyze walking speed as a dimensionless variable^49^ and thus facilitate comparisons among individuals with different leg lengths, we calculated Froude number (*Fr*) as

2 $$Fr=\frac{{v}^{2}}{gL}$$

where *v* is walking speed, *g* is the gravitational constant (9.81 m/s^2^), and *L* is greater trochanter height during standing.

### Statistical Analysis

All statistical analyses were performed in R^50^. All variables were inspected for normality and for homogeneity of variance between the two groups. ADM, ASI and k_mid_ were log-transformed to achieve normality. To test for differences between groups in each anthropometric (AHI, ASI, BMI), muscle cross-sectional area (AH, FDB, ADM), and kinematic (k_mid_ and θ_max_) response variable we created general linear models in which group identity was included as a fixed factor, and different predictor variables were included in the model as follows: *age* for all response variables, *BMI* for response variables that were not scaled by body mass (AHI and θ_max_), and *Fr* for kinematic variables (k_mid_ and θ_max_). We performed ANOVAs on model variance to test for differences between groups. To test for relationships between muscle cross-sectional area and anthropometric/kinematic variables, as well as ASI and k_mid_, we pooled data between groups and checked that variables were linearly related and fit bivariate normal distributions. In cases where these assumptions were met, we used Pearson’s product moment correlations to test for association, and otherwise we used Spearman’s rank correlation. Alpha levels for all statistical tests were set at 0.05.

To assess the potential effects of physical activity on the variables measured in this study, we summed the self-reported hours walked and hours run per day to create a physical activity (PA) variable. PA was poorly matched between groups, and not normally distributed within groups. Thus, we conducted a matched sample analysis by comparing the subset of minimally and conventionally-shod participants who had overlapping PAs (1–3.9 hours walked/run per day), and used Wilcoxon Rank-Sum tests to test for differences between groups in each of the response variables.

### Data Availability

All processed data analyzed for this study are available as supplementary data. Raw data are available from the corresponding author on reasonable request.

## Results

### Anthropometrics and Muscle Cross-Sectional Area

**Figure 4:**
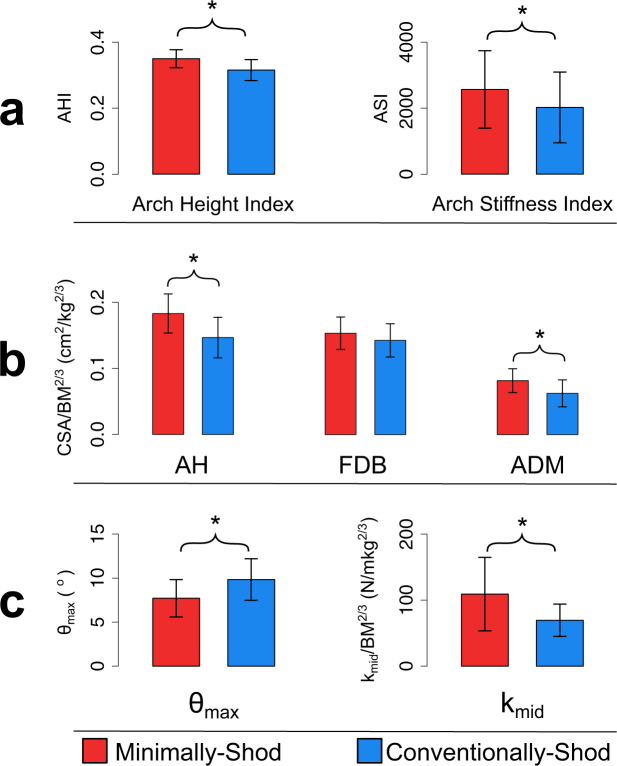
Results of comparisons between minimally- and conventionally-shod individuals. (**a**) Static measurements of arch height index (AHI) and arch stiffness index (ASI). (**b**) Muscle cross-sectional area (CSA) measurements. Note that values are scaled by (body mass [BM])^2/3^. (**c**) Dynamic measurements of maximum arch deformation angle (θ_max_) and arch stiffness (k_mid_). Note that k_mid_ is scaled by (body mass)^2/3^. * denotes statistically significant difference between groups.

Standing arch height as measured by AHI and arch stiffness as measured by ASI were 9% (GLM [General Linear Model]: P < 0.0001, df [degrees of freedom] = 97) and 27% (GLM: P = 0.009, df = 97) higher, respectively, in minimally-shod participants than in conventionally-shod participants (Fig. 4a; Table 1; see Table 2 for GLM results). Based on the AHI cut-off value of 0.297, 31% of conventionally-shod participants (8/26) had low arches, whereas only one of the 75 minimally-shod participants had a low arch (1%). BMI also covaried significantly with AHI (GLM: P = 0.01, df = 97), but BMI did not differ between minimally- and conventionally-shod samples (GLM: P = 0.64, df = 97). Since clear images were not obtainable for all muscles for all participants, we had smaller sample sizes for muscle cross-sectional area comparisons (Table 1). Cross-sectional areas of the AH and ADM in minimally-shod participants were 0.2 cm^2^ and 0.1 cm^2^ larger on average than in conventionally-shod participants, respectively, and these differences were significant after scaling by body size (AH – GLM: P < 0.0001, df = 69; ADM – GLM: P = 0.001, df = 42) (Fig. 4b; Tables 1 and 2). Cross-sectional areas for FDB in conventionally-shod participants were 0.2 cm^2^ larger on average than those in minimally-shod participants, but after scaling for body size FDB was slightly but not significantly larger in minimally-shod participants (GLM: P = 0.2, df = 75) (Fig. 4b; Tables 1 and 2). Of the foot muscle cross-sectional areas measured, only AH was significantly associated with AHI (Pearson’s product-moment correlation [PPC]: P = 0.03, r = 0.26, df = 70). None of the scaled muscle cross-sectional areas were significantly associated with ASI (P > 0.05).

**Table 1:** Means and standard deviations of study variables, and results of ANOVA tests for differences between minimally- and conventionally-shod participants. Tests are carried out on variance from general linear models.

Variable	Minimally Shod		Conventionally Shod		F	df	P-value
*Static*	N	mean ± s.d.	N	mean ± s.d.			
AHI	75	0.35 ± 0.03	26	0.32 ± 0.03	28.6	97	<0.0001
ASI^†^	75	2570 ± 1170	26	2020 ± 1070	7.1	97	0.009
BMI	75	25.7 ± 3.7	26	25.6 ± 3.3	0.2	97	0.64
*Muscle CSA*							
AH^*^	47	2.96 ± 0.51	25	2.77 ± 0.47	20	69	<0.0001
FDB^*^	54	2.45 ± 0.38	24	2.66 ± 0.53	1.7	75	0.2
ADM*^’†^	25	1.24 ± 0.26	20	1.14 ± 0.38	12.7	42	0.0009
*Dynamic*							
θ_max_	30	7.7 ± 2.1	25	9.8 ± 2.4	11.2	50	0.002
k_mid_*,^†^	30	1774 ± 925	25	1304 ± 477	10.4	51	0.002

^*^For general linear models, these variables were scaled by dividing by (body mass)^0.67^.

^†^For general linear models, these variables were logged.

**Table 2:** General linear model coefficients, and results of ANOVA tests on model variance. Reference level for ‘Group’ is minimally-shod participants.

Response	Predictor	Coefficient ± s.e.	F	P-value
AHI	Group	−0.035 ± 0.007	28.6	<0.0001
	Age	−0.0001 ± 0.0003	0.2	0.63
	BMI	0.002 ± 0.001	6.7	0.01
ASI^†^	Group	−0.11 ± 0.04	7.1	0.009
	Age	−0.002 ± 0.002	0.9	0.34
BMI	Group	−0.4 ± 0.85	0.2	0.64
	Age	−0.05 ± 0.04	1.7	0.2
AH*	Group	−0.03 ± 0.01	20	<0.0001
	Age	0.0003 ± 0.0004	0.7	0.41
FDB*	Group	−0.008 ± 0.006	1.7	0.2
	Age	0.0003 ± 0.0002	1.3	0.27
ADM*,^†^	Group	−0.12 ± 0.03	12.7	0.0009
	Age	0.0001 ± 0.002	0.01	0.93
θ_max_	Group	2.09 ± 0.63	11.2	0.002
	Age	0.005 ± 0.04	0.01	0.89
	BMI	0.06 ± 0.08	0.6	0.44
	Froude	−7.91 ± 11.33	0.5	0.49
k_mid_*,^†^	Group	−0.15 ± 0.05	10.4	0.002
	Age	0.0005 ± 0.003	0.04	0.84
	Froude	−0.49 ± 0.87	0.32	0.58

^*^For general linear models, these variables were scaled by dividing by (body mass)^0.67^.

^†^For general linear models, these variables were logged.

### Kinematics and kinetics

**Figure 5:**
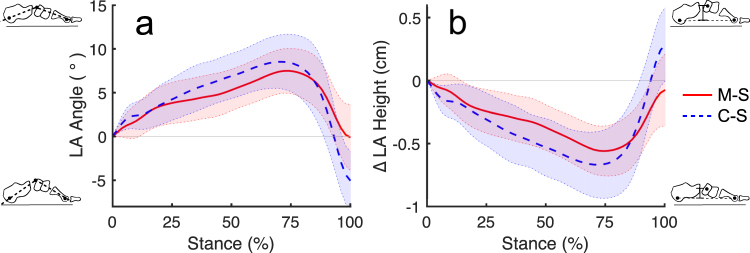
Average foot kinematics during stance phase. (**a**) LA angle and (**b**) Δ LA height during in minimally-shod (M-S; solid line) and conventionally-shod (C-S; dashed line) participants. Shaded regions represent ± one standard deviation. 0° and 0 cm height are defined by marker positions at foot strike.

Conventionally-shod participants had 12% longer legs on average and 7% faster average walking speeds (1.04 ± 0.13 m/s) than minimally-shod participants (0.97 ± 0.18 m/s), but participants from both groups walked with identical average Froude numbers (0.12 ± 0.03), indicating dynamic similarity. For both minimally and conventionally-shod participants, LA height dropped gradually following touchdown until reaching its lowest point around 75% of stance (Fig. 5b). Thereafter, LA height rapidly increased, reaching approximately the same height at lift off as at touchdown in minimally-shod participants, and a slightly greater height than at touchdown in conventionally-shod participants. LA angle changed inversely with LA height, but otherwise followed a nearly identical pattern during stance (Fig. 5a).

Average θ_max_ was 27% higher in conventionally-shod than minimally-shod participants (GLM: P = 0.002, df = 50) (Fig. 4c; Tables 1 and 2). Midstance arch stiffness, k_mid_, was 470 N/cm higher in the minimally versus conventionally-shod participants, a difference that remained significant after scaling by body mass and logging (GLM: P = 0.002, df = 51). Froude did not covary significantly with either variable. k_mid_ was not significantly associated with participant ASI (PPC: P = 0.6, r = −0.07, df = 54). Scaled AH was negatively associated with θ_max_ (PPC: P = 0.001, r = −0.46, df = 44), and positively associated with scaled and logged k_mid_ (PPC: P = 0.03, r = 0.31, df = 44). Scaled FDB and ADM were not significantly associated with either kinematic variable (P > 0.05).

### Effect of Physical Activity

For the PA-matched comparisons, 10 conventionally-shod and 16 minimally-shod participants had PAs between 1 and 3.9 hours/day (Table 3). Within these subgroups, minimally-shod participants were 14 years older on average than conventionally-shod participants (68.8 ± 12.1 *vs*. 54.8 ± 9.9 years; WRS [Wilcoxon Rank-Sum]: P = 0.01, df = 27), and had slightly but not significantly higher BMI (WRS: P = 0.08, df = 27). As in the full sample comparison, minimally-shod participants had significantly higher AHI (WRS: P = 0.005, df = 27), larger scaled ADM (WRS: P = 0.02, df = 12), and higher scaled k_mid_ (WRS: P = 0.007, df = 16) than PA-matched conventionally-shod participants. They also had larger scaled AH and FDB, and lower θ_max_, but this difference was not significant (P > 0.05; Table 3). Unlike in the full sample comparison, conventionally-shod participants had slightly higher ASI than minimally-shod participants, but this difference was not significant (WRS: P = 0.9, df = 27).

**Table 3:** Means and standard deviations for study variables in physical activity-matched sub-samples, and results of Wilcoxon rank sum tests between minimally and conventionally shod participants.

Variable	Minimally Shod		Conventionally Shod		W	df	P-value
*Static*	N	mean ± s.d.	N	mean ± s.d.			
AHI	18	0.36 ± 0.03	10	0.31 ± 0.03	150	27	0.005
ASI	18	2470 ± 1097	10	2608 ± 1473	86	27	0.87
BMI	18	27.4 ± 4.3	10	24.5 ± 2.4	127	27	0.08
*Muscle CSA*							
AH*	12	2.91 ± 0.45	9	2.86 ± 0.50	78	21	0.10
FDB*	15	2.40 ± 0.30	10	2.78 ± 0.46	70	24	0.81
ADM*	4	1.08 ± 0.20	9	0.96 ± 0.16	33	12	0.02
*Dynamic*							
θ_max_	7	8.2 ± 1.4	10	9.6 ± 2.5	25	16	0.36
k_mid_*	7	1897 ± 823	10	1178 ± 364	78	16	0.007

^*^For statistical tests, these variables were scaled by dividing by (body mass)^0.67^.

## Discussion

This study compared intrinsic foot muscle size and foot biomechanics in two groups of adult men while controlling for covariates such as age, body size and to some extent physical activity to test the hypothesis that people who are habitually minimally-shod have bigger foot muscles and stiffer feet than people who habitually wear conventional modern shoes. This hypothesis was supported for two of the three intrinsic foot muscles measured: AH and ADM had significantly larger cross-sectional areas in the minimally-shod than conventionally-shod participants, but FDB did not differ significantly between groups. Our prediction that minimally-shod individuals would have stiffer LAs than conventionally-shod individuals was also supported. We measured LA stiffness both statically as the arch stiffness index (ASI) as well as dynamically at midstance during walking (k_mid_), and for both variables found significantly higher values for the minimally-shod participants. Maximum LA angles (θ_max_) were also significantly higher in conventionally-shod participants, indicating that their arches deformed more during walking than did those of the minimally-shod participants. We predicted that these static and dynamic indicators of LA stiffness would be correlated with intrinsic foot muscle cross-sectional area. While AH was significantly associated with both dynamic LA measurements (θ_max_ and k_mid_), it was not associated with ASI, and the cross-sectional areas of the other two intrinsic foot muscles were not associated with any of the stiffness variables or AHI.

The positive correlation between AH cross-sectional area and foot stiffness is likely related to the muscle’s purported role in medial LA stabilization. In static loading experiments, Kelly *et al*.^51^ found that the AH raises the medial LA when electrically stimulated, and several electromyography studies have also found that AH is active when the LA is loaded during the stance phases of walking and running^4,5,38^. All else being equal, greater muscle cross-sectional area should be directly related to greater force production, and therefore it follows that relatively larger AH muscles should increase foot stiffness more under loading. The fact that AH explained only a relatively small percentage of the variance in θ_max_ (24%) and k_mid_ (10%) is not surprising given that numerous aspects of foot anatomy likely contribute to foot stiffness, including bony geometry, ligamentous structures, and extrinsic foot muscles (e.g. tibialis posterior). Although it is unclear why AH cross-sectional area explains a higher proportion of variance in θ_max_ than k_mid_, it is possible that AH activity is more important for stabilizing the LA when it is maximally deformed, which generally occurs in the second half of stance following heel lift (Fig. 4). The lack of association between AH and ASI may be related to the muscle’s near absence of activation during normal standing^4,52^, suggesting that foot stiffness during this behavior is mostly controlled by passive mechanisms such as ligaments and bony geometry.

Unlike AH, neither FDB nor ADM was significantly associated with dynamically stiffer feet during walking. The result for FDB was surprising considering that Kelly *et al*.^51^ found that stimulating this muscle in statically loaded individuals increased LA height, and other EMG studies have implicated the muscle in dynamically stiffening the foot during walking and running^4,5,38^. However, closer inspection of Kelly *et al*.’s^51^ results reveals that back stimulation of FDB causes adduction and inversion between the calcaneus and metatarsals but not significant sagittal plane flexion, suggesting that the muscle does not actively resist vertical compression of the LA. Additionally, the muscle fibers and tendons of FDB run longitudinally from the inferior calcaneus to the lateral digits of the foot, making the muscle less favorably situated to stiffen the medial side of the LA than AH. We also did not find a significant difference in FDB size between minimally and conventionally-shod individuals, despite finding significant differences between groups in static and dynamic measures of LA height and stiffness. Thus, the results of this study throw doubt on a potential role for FDB in stiffening the medial LA.

The lack of association between ADM and medial LA stiffness is not surprising given the muscle’s location on the lateral side of the foot, which makes it poorly situated to serve as a stiffening truss for the LA. However, the larger size of ADM in minimally- versus conventionally-shod participants suggests an important, but currently unknown role for the muscle in foot biomechanics. This muscle could participate in helping to stiffen the lateral midfoot, which has been shown to be relatively compliant during walking in some individuals with low arches^14^. Additionally, unlike most conventional shoes, the sandals worn by the Tarahumara lack restrictive toe boxes, enabling the fifth digit greater mobility and likely necessitating more frequent use of ADM. However, the functional importance of a more mobile fifth digit in locomotion remains to be investigated.

Our findings for foot muscle cross-sectional area complement previous prospective studies on the effects of minimal running shoes (lacking arch support, cushioning, and heel elevation) on intrinsic foot muscle size. Miller *et al*.^29^ and Johnson *et al*.^31^ randomly assigned individuals to wear either minimal or conventional running shoes for multi-week running regimes, and both found that those in the minimal shoe groups developed significantly larger AH muscles than those in the conventional shoe groups, but not larger FDB muscles. Miller *et al*. also found significantly larger ADM muscles and higher LAs in the minimal shoe group post-treatment, whereas Johnson *et al*. did not measure either ADM size or LA height. Using a similar design to these studies, Chen *et al*.^30^ found a significant increase in overall foot muscle volume in their minimal shoe group but not in their conventional shoe group, although they did not measure the sizes of individual muscles. Whereas these prospective studies suggest that adopting minimal footwear can help increase foot strength and LA height, the present study indicates that habitual use of minimal footwear throughout life is related to bigger foot muscles and stiffer LAs. Taken altogether, these studies suggest foot strength is related to the low incidence of flat foot in habitually barefoot and minimally-shod populations^20–24,28,33^, and also suggest the possibility that flat foot can be treated by switching to minimal footwear that does not restrict the natural motion of the foot.

An additional hypothesis this study tested was that static measurements of LA stiffness would be correlated with dynamic LA stiffness during walking. Surprisingly, we did not find a significant association between our static metric, ASI, and our dynamic metric, k_mid_, even though both were significantly greater in minimally-shod versus conventionally-shod participants. The likely explanation is that a single lower extremity experiences roughly twice the ground reaction force during walking compared to normal standing, likely equating to a two-fold increase in compressive forces experienced by the LA. To resist these higher forces, activity of the intrinsic and extrinsic foot muscles is elicited^4,5,38,39^, whereas these muscles are negligibly active during normal standing^4,52^. Thus, we argue that foot stiffness is dependent on muscle activity during walking but not standing, and that disparities in foot stiffness between walking and standing will be related to the strength of muscles such as AH, as well as other intrinsic and extrinsic foot muscles not investigated in this study (e.g. flexor hallucis brevis and longus, quadratus plantae, tibialis posterior, etc.). Because the forces experienced by the foot during walking are greater than those experienced during standing, dynamic arch stiffness is likely to have much greater implications for the prevention of the common disorders associated with flat foot, including plantar fasciitis, knee osteoarthritis, and metatarsal stress fracture^11,16–19^. Dynamic assessments of arch stiffness are thus probably more useful in clinical examinations of lower extremity musculoskeletal disorders.

Following previous studies, we classified participants with AHI scores 1.5 standard deviations below the mean from a large sample of adult males as having a ‘low arch’^6,35,36^, and found that 31% of our conventionally-shod sample had low arches, whereas only one of the 75 minimally-shod participants that we measured had a low arch. There is not a single universally agreed upon definition of ‘flat foot’^12^, and techniques for diagnosing the condition are disputed^15^, and thus we do not assert that all low arched individuals in this study possessed flat foot. Nevertheless, the near absence of minimally-shod individuals with low arches and the relatively high incidence of low arches in conventionally-shod individuals strongly supports previous studies that have reported low rates of flat foot in barefoot and minimally-shod populations^20–24,28^. Body size is unlikely to have been a factor in the differences found in the present study, as our minimally- and conventionally-shod samples had nearly identical average BMIs with similar variances. Furthermore, the positive association between AH cross-sectional area and AHI suggests that small foot muscles may be related to the presence of low arches, and thus by extension the development of flat foot.

This study has several limitations. First, we included only male participants, as we were unable to collect data from enough Tarahumara females. We do not expect that inclusion of females would change the outcomes of our minimally-shod versus conventionally-shod comparisons, but we do recognize a possible difference in foot stiffness between males and females^37^. Thus far, there have been no investigations of sex differences in intrinsic foot muscle cross-sectional area and dynamic foot stiffness, making this an area requiring further research. Another study limitation is that the minimally-shod participants were subsistence farmers who reported considerably more physical activity (PA) hours per day on average than the conventionally-shod participants, all of whom worked in professions that require little to no physical activity. Despite PA being poorly matched between groups, we assessed its possible effects on foot anatomy and mechanics by comparing the study variables between groups in subsamples matched for PA. The results of these comparisons were similar to the full sample comparisons, with minimally-shod participants having significantly larger ADM muscles, higher AHI, and higher k_mid_ values than conventionally-shod participants. Differences between groups for AH and θ_max_ were not significant, but showed patterns that were consistent with the full sample analyses, with AH being higher and θ_max_ being lower in minimally-shod participants. These results suggest that individuals with minimal shoes have stronger, stiffer arches even after accounting for differences in physical activity, corresponding to the results of a previous investigation of minimally- and conventionally-shod Tarahumara^33^. That said, self-reported physical activity levels are subject to error and do not reflect lifetime differences. Prospective studies are needed to better investigate the effects of physical activity on the variables measured in this study.

One final limitation is that we were only able to estimate dynamic LA stiffness at one moment in a walking stride, mid-stance. This is because the pedography platform we used measures only vertical forces, and mid-stance is when the non-vertical components of ground reaction force are closest to zero, as well as when the linear dimension we used to measure LA displacement is roughly parallel to the ground reaction force. However, as Fig. 4 shows, LA displacement peaks in the second half of stance, following heel lift, suggesting that mid-stance may not be the most functionally relevant moment to calculate LA stiffness. Inverse dynamics calculations of intrinsic foot kinetics have revealed that midfoot moments also peak in the second half of stance^2,13^, at a similar time to the maximum LA deformation angles (θ_max_) that we measured in this study. We measured significantly higher θ_max_ in conventionally-shod versus minimally-shod participants, and found a significant negative correlation between this value and AH cross-sectional area. Thus, we expect that foot stiffness at maximum arch deformation will also be correlated with AH size, but testing this idea requires further investigation with equipment that measures three-dimensional ground reaction forces.

## Conclusions

The results of this study support the hypothesis that individuals who habitually wear minimal footwear have LAs that are stiffer both statically and dynamically than those who habitually where conventional modern shoes. Although prospective studies are necessary to confirm hypotheses of causality, these results also lend support to the hypothesis that certain footwear features affect the cross-sectional areas of foot muscles such as AH, and thus affect LA function. The relatively smaller AH and ADM muscles in the conventionally-shod individuals measured here could be related to features in their shoes that immobilize and protect the foot, such as restrictive toe boxes and raised arch supports. Humans have almost certainly been barefoot for most of the species’ existence, and although humans have been wearing minimal footwear for at least a few thousand years^32^, most of the aforementioned features of modern shoes are extremely recent. Thus, given that the human foot evolved to function unshod and more recently in minimal footwear, its biomechanics may not be entirely adapted for modern “conventional” shoes. We hypothesize that modern shoes reduce the role of the foot muscles in maintaining arch stiffness, thereby leading to less growth and possibly even atrophy through disuse. Subsequently, in some individuals when the arch is unsupported, the foot muscles are not strong enough to prevent arch collapse, resulting in flat foot. If correct, this hypothesis explains the relatively low rates of flat foot among barefoot/minimally-shod populations^20–24,28^.

Beyond the need for prospective studies, future research should target other muscles thought to be involved in maintaining arch stiffness (e.g. flexor hallucis brevis, tibialis posterior). There is also a need for data on individuals from populations in which it is possible to control for potential confounding factors such as genetic ancestry and physical activity levels. Additional research is also needed to determine the effects of specific features of modern shoes (e.g. arch supports) on foot biomechanics to isolate those that may influence foot muscle activity. Finally, further work is needed to determine how the use of modern shoes affects foot function at different stages of development. Previous studies have indicated that shoe use early in life while the LA is still forming may be related to a greater risk of developing flat foot^22,28^. However, there is evidence to suggest that strengthening the intrinsic foot muscles can also lead to a higher, stiffer arch in adults^29–31,53^. Thus, future studies should seek to determine if early development of flat foot is related to under-use of foot muscles in modern shoes, and if the use of minimal footwear later in life can help treat the symptoms of flat foot *via* strengthening of the foot muscles.

## Electronic supplementary material

Supplementary Dataset 1
